# Molecular characterization of *Candida dubliniensis* and *Candida albicans* in the oral cavity of drug abusers using duplex polymerase chain reaction 

**DOI:** 10.18502/cmm.4.1.29

**Published:** 2018-03

**Authors:** Parastoo Hassani Abharian, Parvin Dehghan, Peyman Hassani Abharian, Sepideh Tolouei

**Affiliations:** 1 Department of Parasitology and Mycology, School of Medicine, Isfahan University of Medical Sciences, Isfahan, Iran; 2 Department of Cognitive Rehabilitation, Institute for Cognitive Sciences Studies, Tehran, Iran

**Keywords:** Candida albicans, Candida dubliniensis, Drug, PCR, Smoking

## Abstract

**Background and Purpose:**

*Candida dubliniensis* is closely related to the most pathogenic and prevalent yeast, namely *C. albicans.* *Candida *species can opportunistically overgrow in vulnerable individuals and cause a variety of diseases. The current study aimed to identify and isolate *C. dubliniensis *species present in the *Candida albicans *species complex identified in the oral cavity of drug abusers.

**Materials and Methods:**

This study was conducted on 53 strains of *C. albicans* species complex, isolated from the oral mucosa of drug abusers in Isfahan, Iran. DNA extraction was accomplished through boiling procedure. Duplex polymerase chain reaction (PCR) was performed to amplify ITS1-5.8S-ITS2 region using four specific primers. Fungal species were identified based on the difference in the size of the bands created in the Agarose gel.

**Results:**

Out of the 53 isolates under study, 30 (56.6%) and 14 (26.4%) samples were identified as *C. albicans* and *C. dubliniensis*, respectively. In the remaining 9 samples (17%), both types of *Candida *species were confirmed.

**Conclusion:**

The findings of the present study revealed the presence of a noticeable amount of *C. dubliniensis *in the oral cavity of drug abusers. Therefore, the probable presence of this fungus should be considered during the examination of oral infection among this group. To date, no research has directly investigated this issue in Iran.

## Introduction

*Candida dubliniensis* is closely related to the most prevalent and pathogenic yeast, namely* C. albicans*.* C. dubliniensis* was first identified and named as a new species by Sullivan et al. in Dublin in 1995. This species was primarily isolated from oral candidiasis in patients infected with immunodeficiency virus (HIV). However, it has been also observed in individuals with other infections, such as superficial infections and systemic candidiasis. Nowadays, the outbreak of this yeast is on an increasing trend, while its epidemiology has not been exactly specified yet [1].

*Candida* is one of the commensal micro-organisms, which exists in the oral cavity and opportunistically produces oral candidiasis in vulnerable individuals [2]. However, despite the very close phylogenetic relationship between *C. albicans* and* C. dubliniensis*, they share many phenotypic characteristics, yet epidemiological and pathogenic data indicate that *C.albicans* is a far more successful and prevalent pathogen. 

The dissemination of oral infections results in the development of systemic infections in different organs. *C. dubliniensis* is mostly isolated from the oral cavity of immunodeficient patients, particularly the AIDS/HIV-infected one. However, this yeast has a low capability of infection production and colonization [3, 4]. During the recent decades, the outbreak of *Candida *infections has noticeably increased as a result of the modern medical interventions, such as immunosuppressive therapies. 

Diabetes mellitus, immunodeficiency syndrome, AIDS, and addiction are the predisposing factors for oral candidiasis [5]. The most significant etiologic agents of candidiasis include* C. albicans*, *C. dubliniensis*, *C. glabrata, C. tropicalis, C. krusei, C. parapsilosis, C. stellatoidea, C. kefir, *and* C. guilliermondii *[4]. *C. albicans* is recognized as the most prevalent cause of candidiasis infections due to its high pathogenicity and biofilm formation [6]. 

In a study conducted by Gilfillan et al., *C. dubliniensis* isolate from oral cavities showed a higher adherence to human buccal epithelial cells than *C. albicans* isolates, when grown in glucose. However, they showed equal adherence when grown in galactose culture media [7].

There are numerous medicines for the treatment of oral candidiasis. One of the most important medicines, particularly for patients with extreme immune weakness, is azole compound (e.g., fluconazole). Azoles are alternative to topical polyene agents for the treatment of oral candidiasis in most of the circumstances [6, 8].

Another important species that is less commonly isolated as opportunistic pathogens is* C. Africana*, a newly recognized species isolated from the vaginal mucosa. This species is a doubtful yeast, considered as an atypical strain of *C. albicans*. It can be separated from *C. albicans* morphologically and physiologically, but not genetically [9, 10]. The relative resistance of *C. dubliniensis* to azole compounds, particularly fluconazole, highlights the importance of the identification of such a species [11]. 

The very close relationship between *C. dubliniensis* and *C. albicans, *as well as the common phenotypic and occasionally genotypic properties of these two species, restricts their differentiation. Accordingly, these two species are not separable through using conventional methods. There are different phenotypic methods to differentiate these two species. Nonetheless, molecular methods are considered to be more appropriate strategies to fulfill this end because the kits required for phenotypic analysis have low sensitivity and are time-consuming and costly [12]. 

The accurate and unequivocal identification of these two species is readily and quickly credible via molecular methods, such as Duplex PCR [13, 14]. It seems that it is required to perform molecular tests in addition to the conventional methods to obtain a better understanding of the epidemiological role of *C. dubliniensis *in human infections and prevent the development of drug resistance. These tests are performed to differentiate *C. albicans* and *C. dubliniensis*. 

With this background in mind, the present study was conducted to investigate the identification and confirmation of *C. dubliniensis* present in C. *albicans* species complex identified in the oral cavity of drug abusers. The findings of this study could be helpful in informing the health authorities about this issue.

## Materials and Methods

The current descriptive-inferential study was conducted on 53 *C.* *albicans* species isolated from the oral cavity of 83 drug abusers, who referred to the Addiction Treatment Centers of Isfahan, Iran. These isolates had been formerly isolated and identified as *C.* *albicans *species complex by PCR-restriction fragment length polymorphism method using primers targeting sequences in ITS1-5.8S-ITS2 regions of rDNA. 

***Culture***

Fresh cultures of the yeasts were provided from the samples on Sabouraud Dextrose Agar medium (Merck, Germany).

***DNA Extraction***

The DNA of the yeasts was extracted through the boiling procedure [15]. Briefly, a loopful of fresh isolated colonies were transferred to microtubes, containing 100 μL sterile distilled water and incubated at 90^Ο^C for 20 min in a bain-marie. The microtubes were centrifuged at 10,000 rpm for 10 min. Subsequently, the supernatant was transferred to 0.2 mL microtubes to be used for the next steps.

***Duplex-Polymerase Chain Reaction***

Duplex-PCR was performed by means of four specific primers to amplify the region targeting sequences in the ITS1 and ITS2 regions of rDNA [16]. The sequences of the primers used in the reaction were as follows:

CAL F: 5´ TGGTAAGGCGGGATCGCTT 3´

CAL R: 5´ GGTCAAAGTTTGAAGATATAC 3´

CDU F: 5´ AACTTGTCACGAGATTATTTTT 3´

CDU R: 5´ AAAGTTTGAAGAATAAAATGGC 3´

The method was performed by using a total volume of 25 μl for each reaction, containing 12.5 μl premix (10X PCR buffer, 10 mM dNTP, Taq DNA polymerase at 500 Units/100 μL, and 50 mM MgCl_2_; AMPLIQON III, Denmark), 0.5 μl of each primer (each concentrated at 10 pmol) (Cinagen, Iran), and 3 μL template DNA. Furthermore, molecular grade distilled water was added to obtain 25 μL final volume per reaction.

***Polymerase chain reaction process***

**Figure 1:**
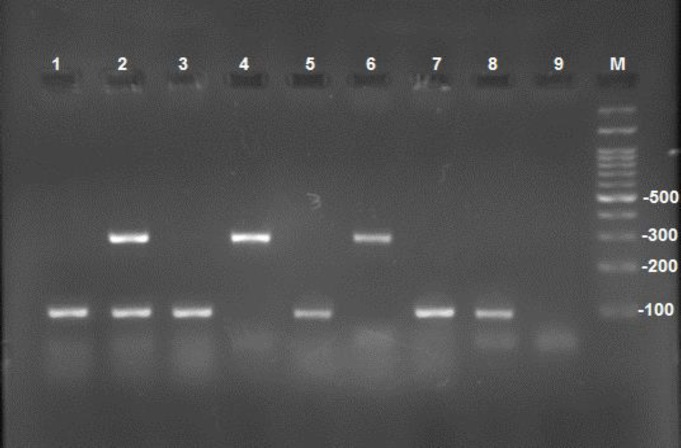
Electrophoresis of duplex-polymerase chain reaction products of some isolates: no. 1, 3, 5, 7, and 8 indicate *C. albicans*; no. 4 and 6 show *C.* *dubliniensis*; no. 2 represents both *Candida *species (i.e., *C. albicans* and *C.* *dubliniensis*); no. 9 signifies the negative control, and M is 100 bp size marker

The PCR included an initial denaturation cycle at 94 ºC for 5 min, followed by 35 cycles 30 sec denaturation at 94°C, annealing at 55°C for 45 sec, and extension at 72°C for 1 min. These cycles were followed by a cycle at 72 ºC for 7 min as the final extension. Electrophoresis was applied in 1.5% Agarose gel to check the PCR products. The gels were examined by the gel documentation system. Through this procedure, *C. albicans* was identified by 100 bp bands, while *C. dubliniensis* was identified by creating 325 bp bands (Figure 1).

***Data Analysis***

The data were compared using univariate Chi-square analysis in SPSS software (version 23 for Windows; SPSS Inc., Chicago, IL, USA). P-value less than 0.05 was considered statistically significant.

## Results

**Figure 2:**
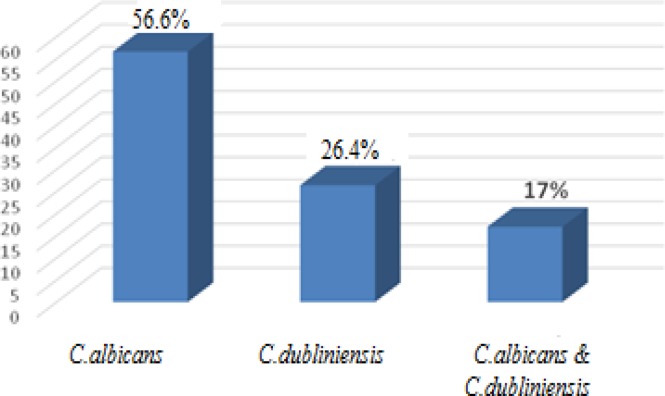
Frequency percentage of *C. albicans* and *C.* *dubliniensis* in *C. albicans* species complex in 53 oral cavity samples of drug abusers

Out of the 53 isolates under study, 30 (56.6%) and 14 samples (26.4%) were identified as *C. albicans* and *C. dubliniensis, *respectively*. *Furthermore, both types of *Candida* were observed in 9 (17%) samples as shown in Figure 2. Table 1 presents the frequency of *C. albicans* and *C. dubliniensis* species isolated from the oral cavities of drug abusers based on the consumed drug. 

***Ethical considerations***

The present study was approved by the Ethics Committee of Isfahan University of Medical Sciences, Isfahan, Iran (reference number: IR.MUI.REC.1395.3.770). Informed consent was obtained from all participants. Furthermore, they were assured about the anonymity and confidentiality terms. 

**Table 1:** Frequency of* C. albicans* and *C.* *dubliniensis* species isolated from the oral cavity of drug abusers based on the type of drug

**Type of drug**	***Candida *** **species**							
	***C. albicans***		***C. dubliniensis***		***C. albicans *** **and ** ***dubliniensis***		**Total**	
	**No.**	**%**	**No.**	**%**	**No.**	**%**	**No.**	**%**
Resin	4	100	---	---	---	---	4	100
Opium	6	54.5	5	45.5	---	---	11	100
Heroin	14	66.7	4	19	3	14.3	21	100
Crack cocaine	2	20	4	40	4	40	10	100
Methamphetamine	17	68	5	20	3	12	25	100
Simultaneous smoking	12	63.2	6	31.6	1	5.3	19	100

## Discussion

Oral candidiasis is one of the most prevalent oral infections, particularly in people with immunodeficiency, like drug abusers or addicts. Addictive drugs are known to change the cell growth and division [17, 18]. It has been documented that the immunity of drug-dependent individuals becomes dysfunctional, hyperstimulated, and suppressed [19]. In this regard, Li et al. showed that methadone enhances the risk of HIV infection [20].

In addition, Morgan et al. suggested that in AIDS patients, *C. dubliniensis* is inherently susceptible to the commonly used antifungal drugs. Furthermore, they revealed that fluconazole resistance occurs in clinical isolates, which can be readily induced in vitro, following exposure to the drug. Therefore, the increased incidence of resistance to antifungal therapy in oral candidiasis results in serious problems [21]. 

According to the literature, *C. albicans* is one of the most common *Candida* species in both patients and healthy people and is generally regarded as the dominant pathogenic species in the susceptible hosts [22, 23]. There are several studies revealing the significant effects of smoking, alone or in combination with other factors, on the oral* Candida* flora; however, few studies have rejected such an effect [24]. 

In the recent years, infections due to species other than *C. albicans* have also increased. *Candida* species may involve any organ systems and greatly contribute to the development of candidemia with a diverse clinical picture. One of the reasons of the higher prevalence of candidiasis in individuals suffering from immunodeficiency is the defects in their defense mechanisms [25]. Therefore, in AIDS patients with candidiasis, the infection is not caused by unique or particularly virulent strains, but probably is due to the host’s defective defense mechanisms [8]. 

Even though *C. dubliniensis* was firstly reported to be observed in the HIV-infected patients, these days, this species is increasingly observed in patients suffering from candidemia, diabetes, and other disorders [13, 26, 27]. In the present study *C*. * dubliniensis* had a high prevalence in drug abusers. *C*. *dubliniensis *might be misdiagnosed with its morphologically related species, namely *C*. *albicans*. *C. dubliniensis *is a relatively new pathogen that is similar to and in very close relationship with *C. albicans*. Nonetheless, it differs from *C. albicans* in terms of epidemiology, physiological properties, and ability to show extensive resistance to fluconazole under laboratory conditions [28].

In the present study, based on duplex-PCR, 30 (56.6%) and 14 (26.4%) samples were identified as *C. albicans* and C*. dubliniensis, *respectively*. *Regarding the other 9 (17%) samples, both types of *Candida *species were observed. Considering the scarcity of reports about *C. dubliniensis* in Iran, the identification of this amount of *C. dubliniensis* is very noticeable. 

Our findings are in line with the results of a study carried out by Hadžić et al. investigating the frequency of *Candida *species in the oral cavity of 60 patients addicted to alcohol and opiates and admitted to Addiction Centers in Bosnia and Herzegovina. In the mentioned study, the frequencies of *C. albicans* and *C. dubliniensis* in the oral cavity of addicted patients were reported as 43% and 23%, respectively [29]. In congruence with our study, Maheshwari et al. studied 82 oral samples of Indian patients infected with HIV and reported the frequencies of 50% and 12.5% for *C. albicans* and *C. dubliniensis*, respectively [30].

In a recent study performed on 120 bronchoalveolar lavage samples obtained from patients with pulmonary disorders in Isfahan, 17 (58.6%) strains were identified as *C. albicans *species complex. In the mentioned study, *C. albicans* with the frequency of 42.9% (n=15) was the most common isolated species, whereas C. *dubliniensis* was identified in 2 cases among the non-HIV patients [16]. Despite using the same molecular method for species identification by the two studies, the discrepancy between our results and those of the mentioned research, observing a low C. dubliniensis frequency, should be due to the difference in the type of disease, underlying disease, and risk factors in the investigated populations. In Abiroo on 186 oral cavity samples obtained from patients with different types of cancers in Kashmir, India, no isolate of *C. dubliniensis *was detected [1]. Inconsistent with our results, in a study carried out by Mohammadi et al., *C. albicans* was the only species observed in the oral samples of diabetic patients [31]. Javaheri et al. reported the *C. albicans *species complex frequency of 42.1% in saliva samples obtained from smokers. In the mentioned study, the implementation of specialized molecular test revealed that all this amount was *C. albicans*. These findings are not in accordance with the results of the current study. This discrepancy could be due to the difference in the type of drug and investigation method [32].

Factors, such as geographical climate, underlying factors, population, sample under study, and research methodology might contribute to the presence of *C. dubliniensis* in the societies and account for the difference between the results of the present research and those of other studies. The consistency of our results with those of the previous studies carried out on the HIV-infected patients (e.g., Maheshwari et al.) can be due to the physiological and immunological similarity of the populations under study. 

According to the literature, some drugs exert a very adverse effect on the immune system. There is a direct relationship between addiction, inefficiency of the immune system, increased pathogen acquisition, and establishment of different templates [34-36]. Despite the significance of this issue, little research has investigated this domain among addicts. Likewise, no study has focused on this area in Iran.

## Conclusion

This study clearly revealed the significant presence of *C. dubliniensis* in *C.* *albicans *species complex in the oral samples of drug abusers. Identification and differentiation of both species result in the achievement of more accurate clinical and epidemiological findings and may cause to prevent from the development of species resistance. It is suggested to conduct further studies regarding *C. albicans *and* C. dubliniensis* using larger groups of addicts throughout Iran to obtain noteworthy results.
